# Impact of macronutrients intake on glycemic homeostasis of preterm infants: evidence from continuous glucose monitoring

**DOI:** 10.1007/s00431-024-05532-4

**Published:** 2024-04-18

**Authors:** Silvia Guiducci, Giulia Res, Luca Bonadies, Federica Savio, Sabrina Brigadoi, Elena Priante, Daniele Trevisanuto, Eugenio Baraldi, Alfonso Galderisi

**Affiliations:** 1 Department of Woman and Child’s Health, University of Padua, Padua, Italy; 2 Department of Developmental and Social Psychology, University of Padua, Padua, Veneto, Italy; 3 Institute for Pediatric Research (IRP), Padua, Veneto, Italy; 4 Department of Pediatrics, Pediatric Endocrinology, Yale University, 333 Cedar Street, LMP3107–06520 New Haven, CT USA

**Keywords:** Preterm infants, Continuous glucose monitoring, Neonatal glucose, Neonatal nutrition, Neonatal hypoglycemia, Neonatal hyperglycemia

## Abstract

Nutritional intake could influence the blood glucose profile during early life of preterm infants. We investigated the impact of macronutrient intake on glycemic homeostasis using continuous glucose monitoring (CGM). We analyzed macronutrient intake in infants born ≤ 32 weeks gestational age (GA) and/or with birth weight ≤ 1500 g. CGM was started within 48 h of birth and maintained for 5 days. Mild and severe hypoglycemia were defined as sensor glucose (SG) < 72 mg/dL and <47 mg/dL, respectively, while mild and severe hyperglycemia were SG > 144 mg/dL and >180 mg/dL. Data from 30 participants were included (age 29.9 weeks (29.1; 31.2), birthweight 1230.5 g (1040.0; 1458.6)). A reduced time in mild hypoglycemia was associated to higher amino acids intake (*p* = 0.011) while increased exposure to hyperglycemia was observed in the presence of higher lipids intake (*p* = 0.031). The birthweight was the strongest predictor of neonatal glucose profile with an inverse relationship between the time spent in hyperglycemia and birthweight (*p* = 0.007).

*  Conclusions*: Macronutrient intakes influence neonatal glucose profile as described by continuous glucose monitoring. CGM might contribute to adjust nutritional intakes in preterm infants.

**Table:** 

**What is Known:** *• Parenteral nutrition may affect glucose profile during the first days of life of preterm infants.*	
**What is New:** *• Continuous glucose monitoring describes the relationship between daily parenteral nutrient intakes and time spent in hypo and hyperglycemic ranges.*

**What is Known:**

*• Parenteral nutrition may affect glucose profile during the first days of life of preterm infants.*

**What is New:**

*• Continuous glucose monitoring describes the relationship between daily parenteral nutrient intakes and time spent in hypo and hyperglycemic ranges.*

Preterm infants are exposed to large glucose fluctuations that could affect long-term neurodevelopment [1, 2] and short-term clinically relevant outcomes as daily growth during the first weeks of life.

Early aggressive parenteral nutrition [3] consists of supplementation of glucose, amino acids, and lipids starting the first hours of neonatal life with a progressive increase of the daily intake generally driven by point-of-care glucose measures and weight change. This strategy seems to be effective at reducing the incidence of postnatal growth failure that, in turn, is associated to growth delay and neurodevelopment impairment [4]. Nevertheless, preterm infants have a low tolerance for parenteral glucose infusion with secondary hyperglycemia [3]. This observation oftentimes leads to a more cautious increase in daily nutritional intakes in those receiving parenteral nutrition with a consequent impact on postnatal growth [3].

Relying on the availability of continuous glucose monitoring (CGM) technology that allows a safe and accurate estimate of time spent in the hypo and hyperglycemic ranges in preterm infants [5, 6], we investigated the relationship between macronutrient intake and glucose homeostasis of very low birth weight infants during the first week of life.

## Methods

### Study design

We conducted a secondary analysis on the cohort of the BabyGlucolight Trial (NCT04347590), a monocentric randomized controlled study investigating the role of CGM on long-term neurodevelopment in preterm infants. The trial was approved by the Institutional Ethics Committee of the University Hospital of Padua (Italy) (4773/AO/19 AOP1813 and 5005/AO/21 AOP2216) and conducted according with the Declaration of Helsinki. Participants were enrolled from March 2020 to June 2022 at the Neonatal Intensive Care Unit (NICU) of the University Hospital of Padua. The enrolment during 2020 was delayed due to the pandemic restrictions in place in our Country.

### Participants

Eligible subjects were preterm infants born ≤ 32 weeks gestational age or with birth weight ≤ 1500 g admitted to the NICU. Written informed parental consent was obtained before recruitment. Exclusion criteria were birthweight < 500 g, congenital malformation, perinatal maternal infections, and albinism. The baseline perinatal characteristics we collected include intrauterine growth restriction (IUGR) (defined by prenatal ultrasound as a reduced growth velocity between two consecutive ultrasound scans for the abdominal circumference or for the estimated fetal weight) [7], gestational diabetes and preterm premature rupture of membranes (pPROM); type of delivery (cesarean section or vaginal), Apgar index, need for endotracheal intubation in the delivery room (ET-DR). Postnatal characteristics were GA, sex, birthweight, and small for gestational age (birthweight < 10^th^ percentile) [8]. We also collected data to describe the course of the first days of life as clinical risk index for babies I score (CRIB I) [9], surfactant administration during the first day of life, episodes of early-onset sepsis (EOS)—within the first 72 h after birth—patent duct arteriosus (PDA) requiring medical treatment (ibuprofen), daily weight change, defined as percentage of weight loss respect to BW, and mortality within the first month of life.

### Glucose monitoring

CGM (Medtronic Guardian 3) was placed on the lateral side of the thigh after adequate disinfection and preceded by containment and analgesia with the use of a pacifier and 0.3 ml 24% sucrose 2 min before the procedure. Sensor was placed within 48 h of life and maintained for 5 days. The device was calibrated as per manufacturer’s instructions, with capillary blood glucose values measured by using an Accu-Chek Inform II glucometer (Roche Diabetes Care, Indianapolis, IN). CGM data were analyzed retrospectively to create a glucose profile for each subject. Mild and severe hypoglycemia were defined as sensor glucose value below < 72 mg/dL (< 4 mmol/L) and 47 mg/dL (< 2.7 mmol/L); mild and severe hyperglycemia were defined for sensor glucose value was above > 144 mg/dL (> 8 mmol/L) and > 180 mg/dL (10 mmol/L). The sensor was manually inserted to prevent accidental insertion in the muscle or injury and calibrated as per manufacturer instructions. Previous studies proved the safety of this sensor in very preterm infants [6, 10].

### Macronutrient’s intake

Daily macronutrients including glucose, lipids, and amino acids were titrated based on day of life and following ESPGHAN guidelines [11]. We collected both parenteral and enteral intakes during the glucose monitoring. Intravenous macronutrients were given through central venous catheter by personalized parenteral nutrition, including dextrose, a mixed oil lipid emulsion (ClinOleic 20% ^®^, 80% olive oil, 20% soybean oil); and an amino acids solution (Primene 10% ^®^). Nutritional intake by enteral feeding was reported if milk volume was above 24 ml/kg/day, considered the upper limit of minimal enteral feeding. Human milk was preferred to preterm formula.

### Statistical methods

The primary outcome was the relationship between macronutrient intake and the percentage of time spent in mild hypo and mild hyperglycemic ranges as recorded by the CGM and daily nutrient intakes (g/kg). Euglycemia represented the time between 72 and 144 mg/dL [4–8 mmol/L] as previously described in very preterm infants [5, 6, 12]. Data were analyzed using SPSS statistical software v 28.0 for Windows (SPSS, Chicago, IL) and Prism 10.0 (GraphPad Software, San Diego, CA). Figures were generated using Prism 10.0 (GraphPad Software, San Diego, CA). Non-normally distributed data were plotted after normal log transformation. We excluded from the analysis those with less than 2 days of CGM data. The daily breakdown of glucose patterns was provided by the day from the CGM start. Macronutrient intakes were reported as g/kg/day. Categorical variables were expressed as numbers (%). Continuous variables were expressed as median and interquartile range (25^th^, 75^th^ centile). The relation between macronutrient intake, birthweight, and time in hypo- and hyperglycemia was analyzed for each variable with a multivariate regression analysis adjusted for GA, sex, and IUGR status. The significance level was set to $\alpha$ < 0.05.

The datasets generated and analyzed during the current study are available from the corresponding author on reasonable request.

## Results

We analyzed data from 30 patients. Participants’ neonatal and maternal characteristics are displayed in Table 1. The total median time of CGM data was 67 h and 57 min (61^h^11^m^; 70^h^23^m^). The median percentage of time in euglycemia was 88.5% (63.7; 97.3) on day 1 without a significant difference across the 3 days of monitoring. The time spent in mild hypoglycemia and hyperglycemia was 0.0% (0.0, 5.4) and 4.0% (0.0, 24.0) for day 1; 0.0% (0.0, 2.6) and 0.0% (0.0, 0.1) for day 2, 0.9% (0.0, 4.9); and 3.5% (0.0, 28.5) for day 3, while the time in severe hypo and hyperglycemia was 0.0% (0.0, 0.0) and 0.0% (0.0, 0.3) for day 1; 0.0% (0.0, 0.0) and 0.7% (0.0, 20.9) for day 2; 0.0% (0.0, 0.0) and 0.0% (0.0, 0.3) for day 3.

**Table 1:** Baseline characteristics (*n* = 30) expressed as median (IQR) or absolute number (%)

**Pregnancy**	
Caesarean delivery, *n* (%)	27 (90)
Maternal diabetes, *n* (%)	6 (20)
pPROM, *n* (%)	9 (30)
Twin pregnancy, *n* (%)	11 (36)
IUGR, *n* (%)	6 (20)

**Delivery**	
Gestational age, weeks, median (IQR)	29.9 (29.1, 31.2)
Male, *n* (%)	13 (43)
Birthweight, grams, median (IQR)	1230.5 (1040.0, 1458.6)
Apgar Index, median (IQR) 1′ 5′ 10′	6 (5, 7) 7 (7, 8) 8 (8, 9)
ET-DR, *n* (%)	2 (7)

**First week of life**	
CRIB I score, median (IQR)	1 (0, 3)
Surfactant <24 h, *n* (%)	18 (60)
PDA, *n* (%)	10 (33)
EOS, *n* (%)	3 (0)
Mortality 1^st^ month of life, *n* (%)	2 (7)

Data are presented as median (25^th^, 75^th^ centile) or number (percentage)

*CRIB* clinical risk index for babies, *EOS* early onset sepsis, *ET-DR* endotracheal intubation in delivery room, *IUGR* intrauterine growth restriction, *PDA* patent duct arteriosus, *pPROM* preterm premature rupture of membranes, *SGA* small for gestational age

**Fig. 1:**
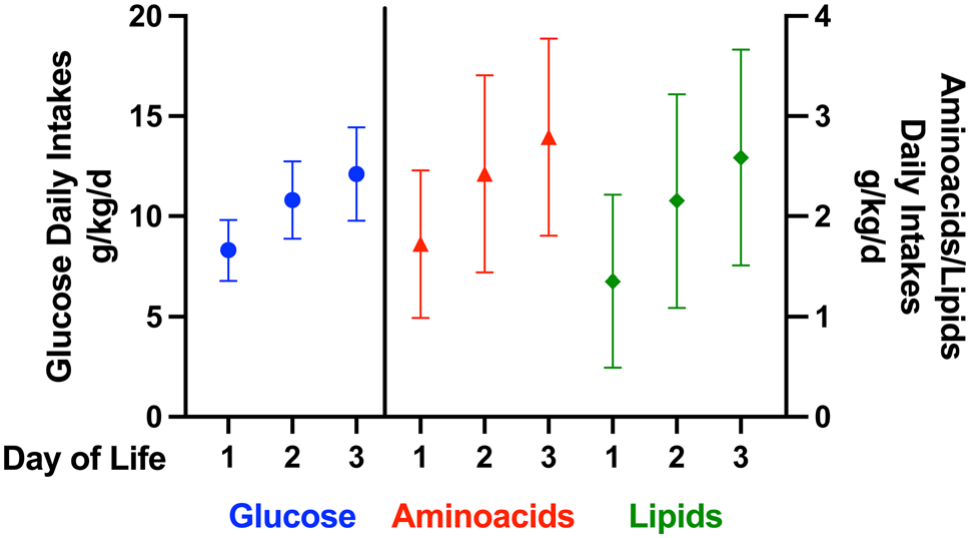
Daily nutrients’ intakes (g/kg/day)

 Figure 1 describes the daily macronutrient intake. As per protocol, glucose intakes were increased by a median of 2.5 g/kg/day, from 8.0 g/kg/day (7.1, 9.4) on day 1 to 12.5 g/kg/day (11.0, 13.7) on day 3, while amino acids intakes from a median of 1.9 g/kg/day (1.4, 2.1) on day 1 to 3.0 g/kg/day (2.0, 3.7) on day 3, paralleled by the lipids intake (from 1.3 g/kg/day (1.1, 1.5) on day 1 to 2.6 g/kg/day (1.7, 3.8) on day 3).

**Fig. 2:**
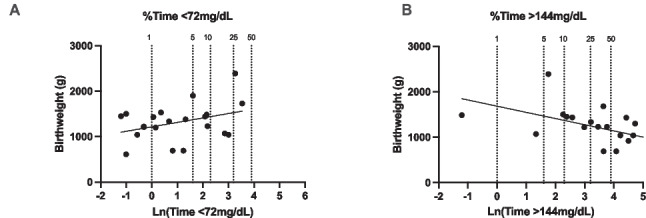
Relationship between the percentage of time spent in mild hypoglycemia (< 72 mg/dL [< 4 mmol/L]) (**A**) and mild hyperglycemia (> 144 mg/dL [> 8 mmol/L]) and birthweight (**B**). Data are naturally log-transformed

 As displayed in Fig. 2B, the birthweight was inversely associated with the time spent in hyperglycemia during the first 3 days of monitoring (*p* = 0.007, *r *= −0.54). This relationship persisted when adjusted for gestational age, sex, and IUGR (*p* = 0.023), while we did not observe a significant relationship between birthweight and the time spent in the hypoglycemic range (*p* = 0.114) (Fig. 2A).

**Fig. 3:**
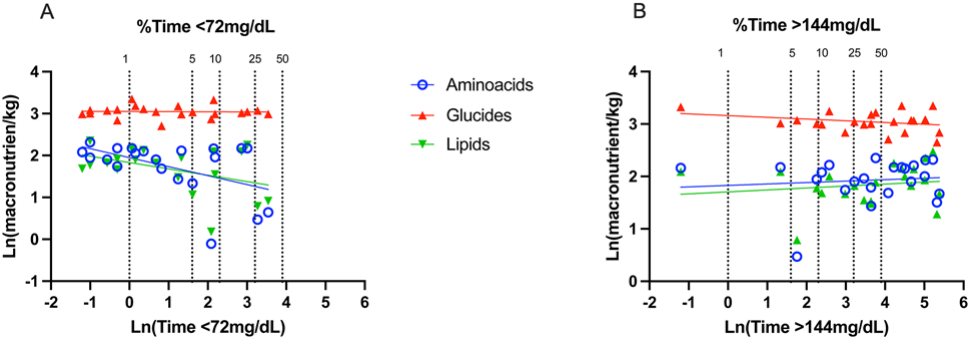
Relationship between the percentage of time spent in mild hypoglycemia (< 72 mg/dL [< 4 mmol/L]) (**A**) and mild hyperglycemia (> 144 mg/dL, [> 8 mmol/L]) and daily nutrient intakes (**B**). Data are naturally log-transformed

 The influence of macronutrient intake on glucose profile is shown in Fig. 3. The total intake of amino acids during the 3 days of monitoring was inversely associated with the time in mild hypoglycemia (*p* = 0.011, *r *= −0.460), and lipids had a similar—though non-significant—relationship with the time in mild hypoglycemia (*p* = 0.066, *r *= −0.268). Glucose intake did not significantly affect the time in the hypoglycemic range (*p* = 0.874) (Fig. 3A). Conversely, the time in the hyperglycemic range over the first 3 days was associated with daily lipids intakes (*p* = 0.031, *r* = 0.238) (Fig. 3B) but not by glucose (*p* = 0.517, *r* = 0.122) and amino acids intakes (*p* = 0.357, *r* = 0.173).

## Conclusion

This study demonstrates the relationship between macronutrient intake and glucose homeostasis during the first days of life of very low birth weight infants wearing a CGM. Higher amino acids intake is associated with reduced time spent in the hypoglycemic range, while greater lipids intake increases the exposure to hyperglycemia. Birthweight remains a main determinant of neonatal hyperglycemia during the first week of life, confirming the increased risk for hyperglycemia in those with the lowest birthweights [13].

Glucose intake itself did not have a major effect on glycemic homeostasis. Although the relationship between daily glucose intake and neonatal glycemia remains controversial [13], herein we proved that glucose instability in preterm infants receiving parenteral nutrition is not exclusively related to parenteral glucose intake while is largely impacted by the amount of amino acids and lipids. Lipid and amino acid intakes largely impact insulin sensitivity, which is in turn a major determinant of hypo and hyperglycemia in the first days of birth of preterm infants, explaining this apparent contradiction. Transient glucose fluctuations are mainly due to procedures and the multiple stressors occurring during the first days of life and directly impact insulin sensitivity. We hypothesize that lipid and amino acid intakes, in turn, influence glucose fluctuations by directly acting on insulin sensitivity.

Our results were aligned with most studies describing a reduction of the hypoglycemic events with higher amino acids intake—between 2.5 and 4 g/kg/day [14, 15].

The main mechanism to support the effect of parenteral lipids infusion on hyperglycemic risk seems to be the stimulation of gluconeogenesis through two components of the lipids infusion, glycerol and free fatty acid (FFA) [16].

The major novelty of our study stands in the use of CGM technology that provides a comprehensive load of data on the actual exposure to hypoglycemia and hyperglycemia rather than point-of-care measures of blood glucose. Due to the large daily variability of glucose during the first days of life in preterm infants, we consider CGM a more informative tool than the point-of-care measures to describe the metabolic homeostasis of preterm infants as well as the effect of nutritional interventions. The accuracy of this tool in preterm infants was warranted by the regular use of calibrations as per manufacturer instructions and has been previously validated by other groups [6, 10, 17]. Previous studies investigating the influence of nutritional intakes on glycemic homeostasis used intermittent glucose tests, while the use of continuous glucose measures unveiled that clinically silent hypoglycemia and hyperglycemia are associated with worse developmental outcomes in childhood [18, 19].

The main limitations of the study are the small sample size and the monocentric design. Even if CGM data were prospectively collected, they were retrospectively analyzed; therefore, we cannot infer a causative relationship between the nutritional intake and glucose control. Moreover, we assessed total macronutrient intake without considering the route of administration that may influence the impact on glucose homeostasis. However, the parenteral amount was more than 60% of the total intake for the entire cohort, thus making it unlikely that the enteral intakes may have a major effect on glucose homeostasis.

CGM appears as a promising tool to individualize nutritional strategies in preterm infants during the first weeks of birth.

## Data Availability

Data is provided within the manuscript or supplementary information. Additional data can be requested to the corresponding author.
